# How We Think about Temporal Words: A Gestural Priming Study in English and Chinese

**DOI:** 10.3389/fpsyg.2017.00974

**Published:** 2017-06-20

**Authors:** Melvin M. R. Ng, Winston D. Goh, Melvin J. Yap, Chi-Shing Tse, Wing-Chee So

**Affiliations:** ^1^Department of Educational Psychology, The Chinese University of Hong KongShatin, Hong Kong; ^2^Department of Psychology, The National University of SingaporeSingapore, Singapore

**Keywords:** time, gestures, priming, metaphor, cross-linguistic comparison

## Abstract

Spatial metaphors are used to represent and reason about time. Such metaphors are typically arranged along the sagittal axis in most languages. For example, in English, “The future lies ahead of us” and “We look back on our past.” This is less straightforward for Chinese. Specifically, both the past and future can either be behind or ahead. The present study aims to explore these cross-linguistic differences by priming auditory targets (e.g., tomorrow) with either a congruent (i.e., pointing forwards) or incongruent (i.e., pointing backwards) gesture. Two groups of college-age young adult participants (English and Chinese speakers) made temporal classifications of words after watching a gestural prime. If speakers represent time along the sagittal axis, they should respond faster if the auditory target is preceded with a gesture indicating a congruent vs. incongruent spatial location. Results showed that English speakers responded faster to congruent gesture-word pairs than to incongruent pairs, mirroring spatio-temporal metaphors commonly recruited to talk about time in their native language. However, such an effect of congruency was not found for Chinese speakers. These findings suggest that while the spatio-temporal metaphors commonly recruited to talk about time help to structure the mental timelines of English speakers, the varying instances in how time is represented along the sagittal axis in Chinese may lead to a more variable mental timeline as well. In addition, our findings demonstrate that gestures may not only be a means of accessing concrete concepts in the mind, as shown in previous studies, but may be used to access abstract ones as well.

## Introduction

### The influence of metaphors on how time is structured in the mind

Metaphors that people use to represent abstract concepts in both written and spoken language may determine the structure of the spatial representations that are generated when conceiving such concepts (Casasanto and Boroditsky's (2008) Integrated Metaphoric Structuring View; Santiago et al.'s (2011) Flexible Foundations theory of Metaphoric Reasoning; Lakoff and Johnson's (1999) Conceptual Metaphor Theory). For example, when talking about time in English, phrases such as “I look *forward* to tonight's dinner” or “We put our past *behind* us” can be used. Speakers may thus possess a sagittal timeline that extends along their front and back. This timeline is shaped by spatial expressions that are used to convey temporal relationships, with the future in front of the individual and the past, behind, paralleling the spatial metaphors used to describe time (e.g., Miles et al., 2010a,b; Christian et al., 2012; Ulrich et al., 2012). Unlike the previously described conceptualization of time where the individual or “ego” is situated along the timeline, an alternative timeline along the sagittal plane has also been proposed where time is conceived as moving from the future to past as though it were a conveyor belt. This is evident in phrases like “The luncheon is after the talk.” Note that the individual is missing in such statements, and time is instead conceived of as a sequence of events, where the “front” is assigned to earlier events and the “back” to later events (e.g., Boroditsky, 2000; Boroditsky and Ramscar, 2002; Gentner et al., 2002).

Radden (2004) argues that time metaphors are arranged primarily along the sagittal axis in the majority of languages, including English and Chinese, around the world. Yu (2012) supported this argument with examples of verbs (e.g., “回顧” *hu*í *gù*^1^; which literally means to look back, to review a past occurrence), nouns (e.g., “前途,” *qián tú*; which literally means the path ahead, future prospects) and proverbs (e.g., “長江後浪推前浪, 世上新人勝舊人,” *cháng jiāng hòu làng tu*ī *qián làng, shì shàng x*ī*n rén shèng jiù rén*; *like the Yangtze River in which the waves behind drive those ahead, so does the newer generation, represented by the waves ahead, surpass the old, represented by the waves behind*) that exist in the Chinese lexicon. Corpus studies (e.g., Chen, 2007) have also found higher frequencies of sagittal metaphors as compared to vertical ones.

**Figure 1:**
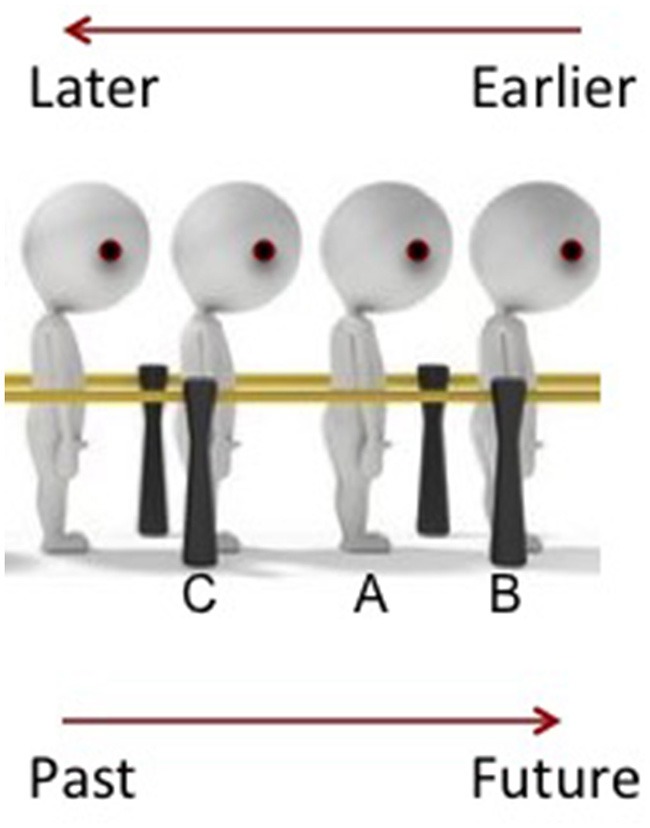
Illustration of a framework proposed by Yu (2012). (A–C) refer to the individual of interest, the person who arrived earlier at the line and the person who arrived at a later point in time.

Yu (2012) argues that it is not sufficient for temporal events to have a “front,” which corresponds to events that took place before it, and a “back,” where events that are yet to occur are situated. It is important for an individual to be located on the timeline as well, with the space in front representing a destination that is to be reached at a future point, and the space behind representing the path taken to reach the present point. To illustrate this in greater detail (see Figure 1), imagine that you (indicated by the person “A”) are part of a line of people queuing up for tickets to an attraction. The person in front of you, “B,” came earlier than you; In other words, he came before you. Likewise, the person behind you, “C,” arrived at the queue at a point in time later than you. Mapping this onto a temporal timeline, if “Person A (you)” were substituted for a temporal event, events that occurred at an earlier point are indeed placed in front, while those that occur at a later point are situated behind. However, note as well the direction that everyone is facing and the destination that everyone is heading toward; for both you, and everyone else in the queue, the destination - the ticket counter, for example, is in front of you. In other words, if a person is situated on the timeline itself, their “future” - the path leading toward the counter, is in front of them, while their “past” - the path they have taken leading to their present point, lies behind them. As such, “past” or “earlier” can be both in front of and behind the individual.

Some researchers have argued that the time metaphors in the case of Chinese are not as straightforward as in English. Linguistic analyses reveal instances in speech and writing in which the speaker or writer faces the past, while the future lies behind (e.g., Alverson, 1994; Ahrens and Huang, 2002). For example, the underlined word of the phrase, “前,年” *qián nián*; which literally means “the year before last,” refers to a space that is in front of the individual. This agrees with observations that Chinese culture tends to be past-oriented with a strong emphasis on the ancestors that came before an individual (Zhou and Fan, 2015). This is especially the case given that whether individuals conceptualize the future or the past as in front of them depends on how future- or past-oriented their culture is (de la Fuente et al., 2014). Other researchers have also suggested that the Chinese tend to adopt a time-moving system (in which the “front” is assigned to an event that took place in an earlier time point; Gentner et al., 2002) when talking about time, thereby making it the dominant representation of time for speakers of the language (Tai, 1993; Alverson, 1994; Ahrens and Huang, 2002; Dong, 2004).

While previous studies may have found similarities in the use of spatial-temporal co-speech gestures along the lateral plane (see Núñez and Cooperrider, 2013 for a comprehensive review), there is little consensus on how Chinese speakers represent timelines in their language, especially along the sagittal plane. The question of interest is how these speakers structure temporal concepts in their mind and whether their timelines along the sagittal plane differ from those of English speakers.

### Gestures as a means to reveal how time is structured

The present study aims to investigate whether English and Chinese speakers would structure timelines in their minds differently. To address this issue, we adopted a cross-modal priming paradigm, with gestures as primes, and binaural auditory tokens as targets. Gestures are speech-associated movements of the hand or arm made to convey ideas and thoughts or to emphasize speech (Kendon, 1983). One study has shown that English speakers gesture forward for future events and backward for past events (Casasanto and Jasmin, 2012).

Using gesture primes may allow us to access temporal concepts in an individual's mind because they provide meaningful information (as participants are able to interpret the message communicated from the gesturer's perspective and hence yoke the axis of interest to the gesturer). Previous studies demonstrated that iconic gestures could prime semantically related concepts (e.g., Wu and Coulson, 2007; Yap et al., 2011). Iconic gestures simulate referents via the motion or shape of the hands. For instance, a speaker says “The bird flew away” while making a flapping motion with their hands to convey information about the bird's actions or attributes. Previous findings have shown that participants responded faster to words (e.g., bird) preceded by semantically related gestures (a FLYING gesture) than to words preceded by unrelated gestures (a DRIVING gesture) (Yap et al., 2011). Similarly, previous research has also found priming effects when gestural primes are accompanied by speech (though of a weaker magnitude as compared to a gesture only condition; So et al., 2013).

The primes in the present study are metaphoric gestures, which represent abstract concepts. For example, a gesture toward the space behind the speaker represents an event that occurred in the past. This allows us to capture a true “front” or “back,” compared to previous studies that had response points in front of the participant that were either nearer or farther relative to their bodies (e.g., Fuhrman et al., 2011). Binaural auditory tokens comprising isolated temporal words that are either past- or future-related were chosen as a means to probe temporal concepts. These stimuli have been found to be as effective as visual stimuli in doing so (e.g., Ouellet et al., 2010).

Earlier studies that have attempted to investigate the differences in how English and Chinese speakers represent time often did so using response congruency (e.g., Fuhrman et al., 2011). In their experiments, researchers place the incongruency at the response stage and focus on how incongruencies (or congruencies) between response and stimuli might hinder (or facilitate) participants' responses. Specifically, participants respond in a manner that is either congruent with how time is represented (left button for past, right button for future) in some trials, or incongruent (right button for past, left button for future) in other trials. However, constraining participants to a particular response format may predispose them into adopting transient frames that are then used to represent time. For example, requiring participants to respond in a congruent, and then an incongruent manner along the lateral plane would emphasize and predispose participants into arranging temporal concepts along this plane. In short, participants may have been primed to represent time in certain ways by virtue of the paradigm used rather than by temporal representations in the mind. In the present study, temporally-related targets will be preceded by gestural primes indicating a space either in front of or behind the gesturer. If the time-related target is semantically connected to the sagittal plane, it should speed up the processing, and hence, responses made to the former. For example, if the target “昨天” (“*zuó tiān*; yesterday”) were to be preceded by a gesture indicating the space in front of the gesturer, responses made to that target should be facilitated compared to a gesture indicating the back since past concepts are placed in front of the individual and future concepts behind.

We hypothesized that English speakers represent time along the sagittal plane. Therefore, they should respond faster if the auditory target is preceded with a gesture produced along the sagittal plane. For example, for the target, “yesterday,” which is a deictic past-related word, a gesture that indicates the space behind the gesturer should facilitate responses compared to a gesture indicating the front since past concepts are commonly situated behind the individual and future concepts in front when an ego-moving perspective is adopted. As mentioned above, English speakers sometimes adopt a time-moving perspective as well. However previous studies that examined the production of temporal gestures by such speakers in spontaneous, conversational settings (e.g., Casasanto and Jasmin, 2012) or in instances when prompted by an ambiguous temporal phrase (e.g., Lai and Boroditsky, 2013) reveal a preference for an ego-moving perspective, with the future ahead and the past behind. In light of these previous findings, we do not expect that our English speakers would adopt a time-moving perspective when understanding the gestures of another.

Chinese speakers, on the contrary, may or may not display such facilitation along the sagittal plane. Given that spatiotemporal metaphors found in language reflect temporal concepts in the mind (Lakoff and Johnson, 1999; Lai and Boroditsky, 2013), one may expect that the future (and past) is represented both in front of as well as behind the individual, depending on the circumstances faced by a Chinese speaker when thinking about temporal events. Two possibilities were derived: The first possibility is that Chinese speakers would show a reversed pattern as compared to the English speakers, suggesting that their time representations in the mind reflect the past-forward, future-behind metaphors used in addition to the past-oriented cultural roots. If the time-related target is semantically connected to the sagittal plane, it should speed up the processing, and hence, responses made to the former. For example, if the target “昨天” (“*zuó tiān*; yesterday”) were to be preceded by a gesture indicating the space in front of the gesturer, responses made to that target should be facilitated compared to a gesture indicating the back since past concepts are placed in front of the individual and future concepts behind. The second possibility, on the other hand, is that Chinese speakers possess a more flexible mental representation of time with the future (and past) both potentially being in front of, as well as behind the individual. If so, the expected results would become less clear. Both possible representations (past forward, future behind vs. future forward, past behind) may be active at the same time within the same participant, thereby canceling out any observable effects of congruency along the sagittal axis.

## Method

### Participants

Forty-three English-speaking participants from the National University of Singapore participated for course credit or payment. A further 43 Chinese (Cantonese)^2^-speaking undergraduates from the Chinese University of Hong Kong participated for payment for the Chinese version of the experiment. While the participants were bilinguals (the English-speaking participants knew Mandarin and the Chinese-speaking participants knew some English), they were native speakers of English and Chinese, respectively, and reported a dominant mastery of their native language over their second language. All participants had no speech or hearing disorders at the time of testing. Participants' ages ranged from 18 to 23 and 58% of them were female. Participation occurred with informed consent and experimental protocols were approved by each institution's respective Institutional Review Board.

### Design

A 2 (Group: English vs. Chinese speakers) × 2 (Congruency: congruent vs. incongruent prime) mixed design was used, with group being the between-subjects variable and congruency, the within-subjects variable. Congruency was determined with respect to the gesturer, so a video where the gesturer points toward the back is considered to be congruent if it was paired with a past-related word, and incongruent if paired with a future-related one for the English group. On the other hand, for the Chinese group, a point toward the back would be considered as congruent when paired with a future-related word, and incongruent if paired with a past-related one.

### Materials

For the English stimuli, time-related words were chosen from the online Oxford Dictionaries (2015). Ten students from the same population sample, but who did not take part in the main experiment, rated how past- or future-related the words were on a 5-point Likert scale (1 = strongly past-related and 5 = strongly future-related). A final set of 40 past- and 40 future-related words, was chosen from words that were rated as either past- (<3) or future-related (>3). Interrater reliability was high and there was high agreement between raters on whether a word was past- or future-related, Cronbach's α = 0.967.

The stimuli for the Chinese experiment comprised a translated subset of the English words. These translated words were likewise normed in the same manner by students from the same population sample as the Chinese group, but who did not take part in the main experiment. A final set of 22 past- and 22 future-related words were chosen, and interrater reliability was high, suggesting high agreement between the raters as to whether a word was past- or future-related, Cronbach's α = 0.977. The reduced stimulus set was a consequence of some words losing a great part of their temporal flavor when translated from English. For example, the Chinese counterpart to the English word, “vintage” (“酿酒”; *niàng jiǔ*) refers almost exclusively to a wine. Unlike in English, where “vintage” might be paired with words like “*car*” or “*record*,” this is not so for its Chinese translation equivalent. All stimuli were spoken by a linguistically trained female speaker and recorded digitally in 16-bit mono, 44.1 kHz, wav format. These files were then digitally normalized to 70 db, thereby ensuring that all tokens had similar overall root-mean-square amplitudes. Log Subtitle word frequencies (LogSUBTLWF) were obtained for both the Chinese (Cai and Brysbaert, 2010) and English words (Balota et al., 2007). A one-way ANOVA reveals no significant differences in LogSUBTLWF between the English and Chinese words [*F*_(1, 119)_ = 2.59, *p* > 0.1]. Word characteristics are summarized in Table 1 and the stimuli are listed in the Appendix.

**Table 1:** Mean temporal characteristics and duration of the past- and future-related words.

	**Past-related words**		**Future-related words**	
	***M***	***SD***	***M***	***SD***
Temporality rating (Chinese stimuli)	1.59	0.14	4.17	0.26
Temporality rating (English stimuli)	1.58	0.39	3.99	0.24
Word duration (Chinese Stimuli; ms)	956.36	165.22	997.68	106.40
Word duration (English Stimuli; ms)	853.38	166.27	821.68	165.16
LogSUBTLWF (Chinese stimuli)	2.58	0.98	2.80	0.79
LogSUBTLWF (English stimuli)	2.15	0.96	2.63	1.03

**Figure 2:**
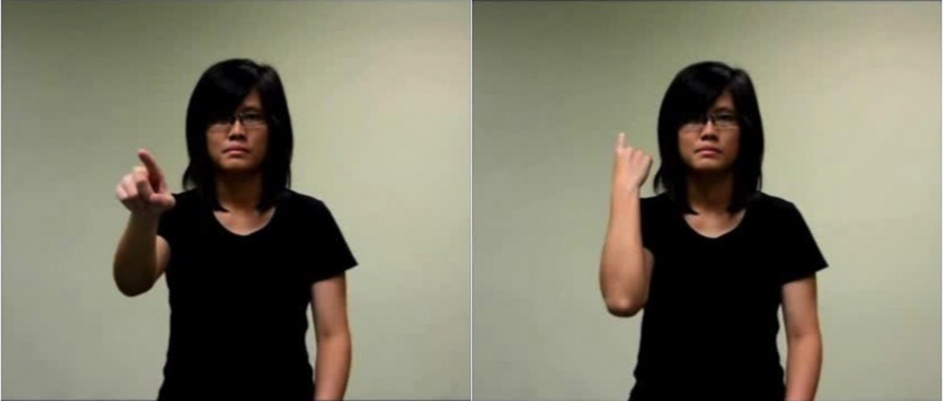
Snapshot of the forward-pointing and backward-point gestures. The individual in the picture above is that of a research assistant employed by our lab and consent was obtained from this individual for the publication of the image.

Counterbalancing was done across participants to ensure that all targets were preceded equally often by congruent and incongruent gestural primes. Two gesture video clips were paired with either congruent or incongruent temporal words. These clips were silent videos of a female actor facing the viewer and pointing either backwards or forwards along the sagittal axis. The video included the actor's face. One video was made for each gesture of interest, with an emphasis on its stroke (Figure 2 shows snapshots of the gestural stimuli used. Please refer to Supplementary videos 1 and 2 for the pointing forwards and pointing backwards clips respectively).

### Procedure

**Figure 3:**
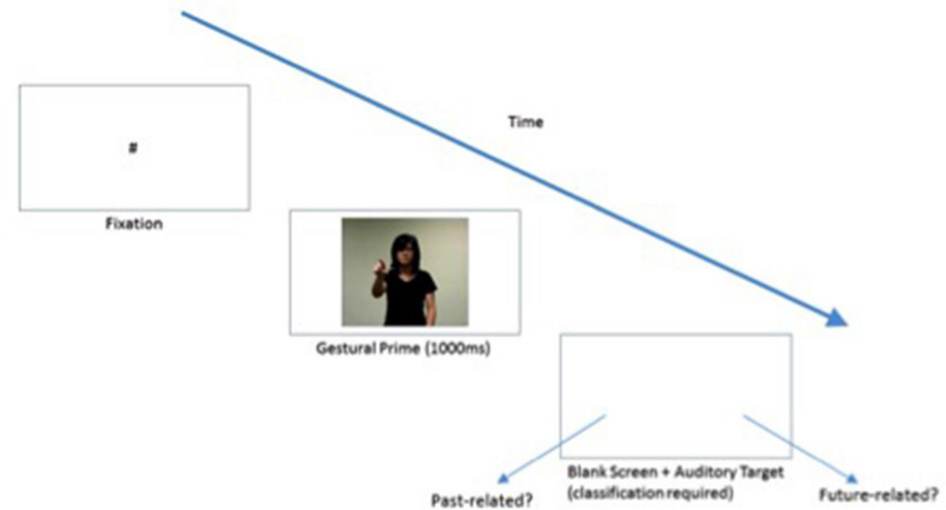
Basic outline of each trial. The individual in the picture above is that of a research assistant employed by our lab and consent was obtained from this individual for the publication of the image.

E-Prime 2.0 (Psychology Software Tools, Pittsburgh, PA^3^) was used for stimulus presentation and response recording. Participants were seated in front of a 17-inch screen and instructed to make a temporal judgment, as quickly and accurately as possible, of the word using two laterally adjacent buttons located at a central location. The left and right buttons labeled *Past-related* (or 與過去有關 yǔ guò qù yǒu guān) and *Future-related* (與未來有關 yǔ wèi lái yǒu guān), respectively in the language of each group. Each trial began with a gesture video clip lasting 1,000 ms, followed immediately by an auditorily presented temporal word to be judged as either past- or future-related. These temporal words were binaurally played through headphones at approximately 70 dB SPL. Response time (RT) was measured from the onset of the auditory target. All trials were conducted in the native language of each group. See Figure 3 for the basic outline of each trial. Prior to the actual experiment, 10 practice trials were administered for familiarization. This was followed by experimental trials randomized for each participant across 4 blocks of 40 trials each (for the English-speaking participants) or 2 blocks of 44 trials each (for the Chinese-speaking participants). The inter-trial interval was set at 500 ms, with a short break after each block. The testing setup was identical in both universities.

## Results

**Figure 4:**
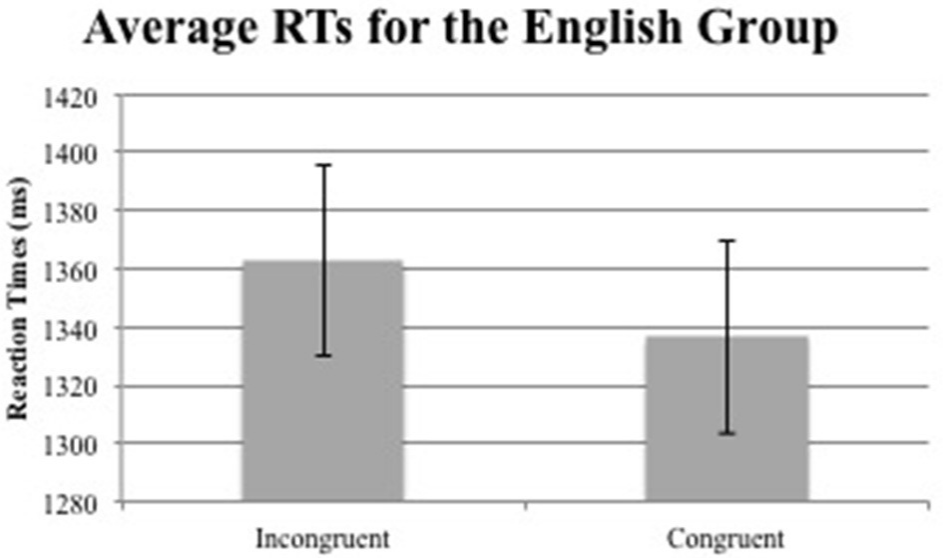
Average RTs for the English group (error bars indicate 1 standard error above and below the mean).

**Figure 5:**
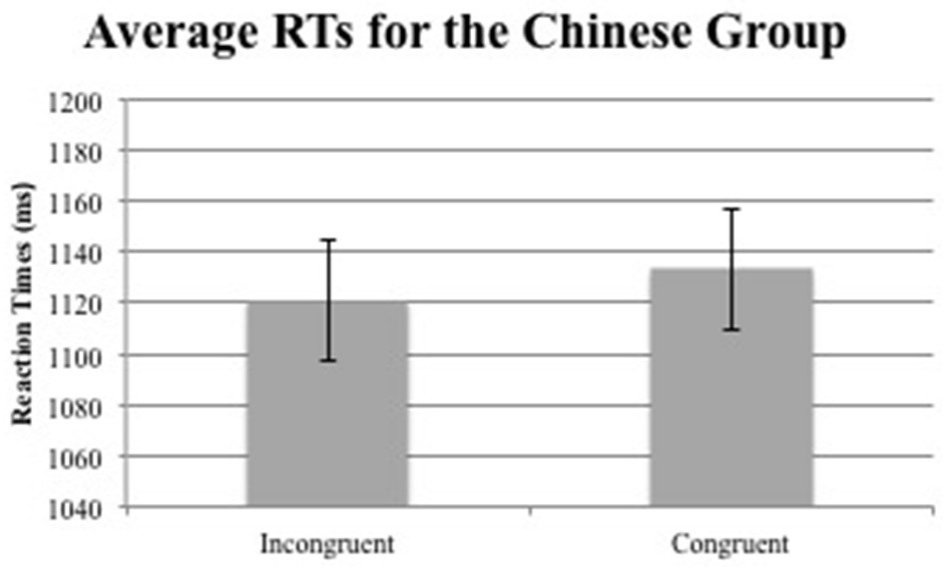
Average RTs for the Chinese group (error bars indicate 1 standard error above and below the mean).

For the RT data, only correct judgments with RTs more than 200 ms and less than 3,000 ms were included in the analyses. Then, the overall mean and *SD* of each participant's RT was calculated and trials with latencies 2.5 *SD*s above or below each participant's mean RT were removed. These trimming criteria resulted in a removal of 4.50% of accurate trials. Overall accuracies were all very high (*M* = 0.93, *SD* = 0.07). The mean RTs for the English and Chinese groups are summarized in Figures 4, 5 respectively, and the exact numbers of the means and standard error (SE) of each group in Table 2.

**Table 2:** Mean RTs and SE for English and Chinese groups.

	**Incongruent pairs**		**Congruent pairs**	
	***M***	***SE***	***M***	***SE***
English	1362.82	34.41	1336.47	32.52
Chinese	1120.79	24.79	1133.12	23.43

The statistical analysis tool R (R Core Team, 2012) and packages, *lme4* (Bates et al., 2014) and *lmerTest* (Kuznetsova et al., 2016) were used to perform a linear mixed effects analysis of the relationship between a participant's response time and the factors of language and congruency. Linear mixed analyses were done to take into account the random effects of both items and subjects by including them into the model as random factors. In addition, it allowed for the control of the varied articulation duration of each item. A log transformation was conducted on the RTs prior to analysis (Baayen et al., 2008). An interaction between congruency and language background was expected, and as such, an interaction term was included in to the model as a fixed effect. For random effects, intercepts were created for individual participants and auditory tokens. The full model is presented in Table 3. A significant interaction was determined (*t* = −2.35, p = 0.019).

**Table 3:** Results of the mixed-effects analysis for both English and Chinese groups combined.

**Overall model**			
**Random effects**		**Variance**	**SD**
Subject	Intercept	0.005020	0.07085
Item	Intercept	0.002124	0.04608
Residual		0.016637	0.12898
**Fixed effects**	**Estimate**	**SE**	***t*****-value**
Intercept	2.931e + 00	2.889e-02	101.454^***^
Congruency	−1.468-e03	3.306e-03	−0.444
Group	1.056e-01	1.872e-02	5.644^***^
Token duration	1.740e-04	2.997e-05	5.805^***^
Congruency^*^Group	−1.553e-02	6.612e-03	−2.349^*^

* *p < 0.05*,

*** *p < 0.001*.

Follow-up analyses were conducted to determine the nature of this interaction by focusing on each group at a time. Congruency was found to affect logRT (*t* = −2.21, *p* = 0.027), reducing it by −9.254E-03 ± 4.183E-03 (standard errors) for the English group, but not the Chinese group (*t* = 1.21, *p* = 0.22). The results of each analysis are presented in detail in Tables 4, 5 for the English and Chinese group respectively.

**Table 4:** Results of the mixed-effects analysis for the English group.

**English group**			
**Random effects**		**Variance**	**SD**
Subject	Intercept	0.006055	0.07781
Item	Intercept	0.002849	0.05337
Residual		0.013924	0.11800
**Fixed effects**	**Estimate**	**SE**	***t*****-value**
Intercept	2.971e + 00	3.523e-02	84.333^***^
Congruency	−9.254-e03	4.183e-03	−2.212^*^
Token duration	1.903e-04	3.853e-05	4.938^***^

* *p < 0.05*,

*** *p < 0.001*.

**Table 5:** Results of the mixed-effects analysis for the Chinese group.

**Chinese group**			
**Random effects**		**Variance**	**SD**
Subject	Intercept	0.0038864	0.06234
Item	Intercept	0.0008787	0.02964
Residual		0.0195539	0.13984
**Fixed effects**	**Estimate**	**SE**	***t*****-value**
Intercept	2.931e + 00	4.350e-02	67.370^***^
Congruency	6.269-e03	5.177e-03	1.211
Token duration	1.209e-04	4.272e-05	2.831^**^

** *p < 0.01*,

*** *p < 0.001*.

Effect sizes were determined by calculating Ω^2^ as recommended by Xu (2003). This was found to be 0.43 and 0.22 for the English and Chinese groups respectively. The statistical package *MuMIn* (Bartoń, 2013) was also used to determine the marginal and conditional R^2^ for each group. The marginal and conditional *R*^2^ for the English group was 0.043 and 0.42, respectively. As for the Chinese group, the values were 0.01 and 0.20 respectively.

To rule out the possibility of any temporal words coming across as spatial words (e.g., “after” being seen as referring to something that comes after another and hence being congruent to the space behind an individual), we also conducted item analyses to ensure that these words were unambiguously understood by their temporal interpretations. For the English words, the temporal words were unambiguously interpreted as such and faster responses were made when they were paired with a congruent gesture (1,313 ms) as compared to an incongruent gesture (1,340 ms). For example, the word “after” was responded to consistently faster by participants when paired with a forward point gesture (1,202 ms) as compared to when it was paired with a backward pointing gesture (1,299 ms)^4^. In the case of the Chinese words, there was an absence of any effects of congruency (incongruent, 1,143 ms vs. congruent, 1,144 ms), mirroring the findings above.

Thus, an effect of congruency was found along the sagittal plane indicating that gestures were able to prime temporal concepts that were either congruent or incongruent with the auditory target for the English group. Furthermore, the effect of congruency was found to mirror the spatiotemporal metaphors commonly recruited to talk about time, with the past situated behind the individual and the future, ahead. For the Chinese group, however, no such congruency effect was found.

## Discussion

In the present study, we investigated whether individuals represent time along the sagittal plane. Using our cross-modal priming paradigm with temporal gestures as primes and auditory tokens as targets, we found an effect of congruency along the sagittal axis for English speakers, suggesting that it is recruited for the representation of time in the mind. On the other hand, this effect of congruency was absent for the Chinese speakers.

### Spatiotemporal metaphors found in language influence representation of temporal concepts

Our results support the notion that spatiotemporal metaphors found in language reflect temporal concepts in the mind (Lakoff and Johnson, 1999; Lai and Boroditsky, 2013). The overwhelming abundance of metaphors aligned along the sagittal plane in English (Radden, 2004), coupled with the almost complete absence of lateral spatiotemporal metaphors, would predispose individuals in representing time along the sagittal axis. This is in line with a psycholinguistic point of view that almost all languages in the world associate time along the sagittal axis (e.g., Radden, 2004). Thus, the metaphors speakers use may play a causal role in how people construct temporal representations, consistent with the claims made by recent (Casasanto and Boroditsky, 2008; Santiago et al., 2011; Lai and Boroditsky, 2013) and traditional (Lakoff and Johnson, 1999) theories. Furthermore, these metaphors continue to affect how mental timelines are structured, even in their absence. No spatial metaphors preceded the English stimuli, and yet the English speakers displayed congruency effects in line with how time is conventionally represented along the sagittal axis.

One could argue that given Chinese temporal words frequently contain a directional component (e.g., 回顧, Huígù, to look back, reflect), and that this might have affected the responses of the Chinese group. However, the majority of the Chinese temporal words used in the present study were free from a directional component. Of the 22 past-related and 22 future-related words, only 3 past-related words (之前, zhīqián, prior to, 以前, yǐqián, before, 史前, shǐqián, prehistoric), and 7 future-related words (然後 ránhòu, then, 日後, rìhòu, after that, 此後, cǐhòu, afterwards, 隨後, suíhòu, hereafter, 後續, hòuxù, follow-up, 之後, zhīhòu, following, 向前, xiàng qián, ahead) had some form of spatial component. Omission of these directional words results in a similar pattern of null findings for the Chinese participants. As mentioned above, we were able to rule out a spatial interpretation of the English temporal words as well. Words like “after” were unambiguously considered by the English participants to be a future related word and congruency effects were dominant when paired with a congruent forward-pointing gesture. As such, when prompted by directionally neutral temporal words (as was the case of the English stimuli) and non-directional words (in the case of the Chinese stimuli), the metaphors found in both languages continue to structure mental timelines.

Another possibility is that the Chinese speakers might have shown a reversed preference along the lateral plane as well, thereby influencing the responses made by this group. However, given that the responses were made along the lateral plane (which is orthogonal to the plane of interest, the sagittal one), we do not expect a mapping or rotation of the sagittal plane onto the lateral one, giving rise to this scenario. Furthermore, previous literature suggests that the lateral plane is largely influenced by reading and writing direction (see Núñez and Cooperrider, 2013 for a comprehensive review). Nonetheless, we cannot fully discount the possibility of the scenario described, and future studies can investigate this in greater detail.

The absence of a congruency effect in the Chinese group may be accounted for by Yu's (2012) linguistic framework. A timeline may comprise of temporal events that possess a “front,” where events preceding it are situated, and a “back,” where events that are yet to occur are placed. When an individual is placed on the timeline, however, the space in front becomes a destination that is to be reached at a future point, while the path taken to reach the present point lies behind. However, it is worth noting that Yu's (2012) framework does not capture the nuances of cross-language differences. For instance, Chinese speakers may display less flexibility (and hence, more similar responses) when interpreting ambiguous temporal phrases like “moving forward a meeting by 2 days” as compared to English speakers. While this was not the focus in the present study, given the unambiguous nature of the temporal words, future studies may look into this by incorporating such ambiguous temporal phrases in both languages alongside potentially disambiguating gestures.

At first glance, our findings appear to run contrary to previous studies that have found similarities between Chinese and English speakers in terms of their use of spatial-temporal co-speech gestures along the lateral plane (Núñez and Cooperrider, 2013). However, this is not so as, unlike previous studies, the focus is on the sagittal plane instead of the lateral one. Along the lateral plane, there is little dispute in terms of the directionality: both English and Chinese speakers (in this study, at least) read and write from left to right and would also be expected to gesture similarly given such conventions. It is along the sagittal plane, however, where English and Chinese speakers appear to differ.

In summary, Chinese speakers may possess a more flexible mental representation of time (at least, along the sagittal axis), given the wide variety of metaphors that place the future (and past) both in front of, as well as behind the individual. As such, the Chinese temporal words used may have activated both possible directions that the mental timeline could have taken, consequently resulting in a lack of congruency effect as the two potentially cancel each other out. Future studies might look at production data from interviews to see if Chinese speakers gesture forward or backward when talking about past and future events and if the direction of the sagittal axis is dependent on the metaphor used, the subject matter of the discussion at hand, or perhaps even the smaller units of the words themselves.

It is also possible, however, that the Chinese participants may have simply not activated any spatial representations when making the temporal judgments in the present study. While this is unlikely, given previous experiments (e.g., Lai and Boroditsky, 2013) where, when tasked to point to regions of space in front of them to represent various time points with respect to a space designated as the present, Chinese-speaking participants systematically produced points that corroborated the directional metaphors used. Nonetheless, this possibility may be addressed by adopting a similar method by having participants point (with respect to themselves as the present moment) to place the temporal stimuli used in the present study. For example, we could ask participants where they might point to in order to represent the word, 昨天 (*zuó tiān*, yesterday), if their body represented the present moment.

### Utility of cross-modal priming with gestures as a means to tap spatiotemporal concepts

The present study demonstrates the usefulness of not only the cross-modal priming paradigm as a means to investigate how individuals represent time in the mind, but also the viability of gestures as primes to access temporal concepts in the mind. When we talk, we gesture (McNeill, 1992). Gestures are found to accompany temporal speech (Casasanto and Jasmin, 2012), e.g., English speakers gesture forward for future events and backward for past events. Previous research has also shown that iconic gestures prime semantically related words and concepts (Wu and Coulson, 2007; Yap et al., 2011). Similarly, our study reported that temporal gestures prime temporal concepts, suggesting that these gestures could access temporal representations in an individual's mind. A further benefit is that gestures allow for a meaningful representation of a “forward” and “behind” with respect to the gesturer. It should be noted, however, that there is a degree in the flexibility in using spatial gestures for temporal verbal expressions based on the context. For instance, the temporally ambiguous phrase “the meeting on Wednesday has been moved forward by a couple of days” may be interpreted very differently depending on whether a gesture indicating the space in front or a gesture toward the back is used. Nonetheless, the stimuli used in this study are unambiguously past- or future-related, and such aforementioned flexibility was not a factor of consideration.

The auditory tokens were likewise efficacious in tapping the temporal concepts activated by the temporal gestures. Previous studies have often used visually-presented sentences or phrases to probe temporal concepts, but this choice of stimuli might predispose participants into adopting the frames provided (i.e., from left to right) to represent time during the experiment (Casasanto and Bottini, 2014). The reading of sentences and phrases may also enable participants to reflect on time representations and allow for strategic processes that would then obscure how time is naturally represented. Hence, by having our participants respond as quickly as possible to isolated words, such strategic processes are potentially minimized, allowing underlying temporal representations to be tapped.

The present study also has another implication for future investigations into the mental timeline structure. When a response congruency method is not used, spatial frames that might potentially be transiently adopted to represent time are withheld from individuals. Such findings are partially replicated in Lai and Boroditsky (2013, Study 2) where, when allowed to point to regions in space instead of being constrained along certain modes of response, participants are found to represent time along the sagittal axis as well when asked to arrange events in temporal order. This echoes the point noted by Walker et al. (2014) that the majority of space-time associations observed thus far in response congruency experiments (e.g., Fuhrman and Boroditsky, 2010; Boroditsky et al., 2011) could have partially arisen from the demand characteristics of the experimental paradigm.

### Future directions

Future studies may explore if these findings are specific to gesture or if other spatial stimuli (e.g., arrows) may similarly prime temporal concepts in individuals. The sagittal representation of time has been proposed to be oriented with respect to an individual's sagittal axis, and is grounded in the experience of front-back locomotion that our bodies are accustomed to (Clark, 1973; Lakoff and Johnson, 1999; Radden, 2004). As such, while other spatial stimuli may prime temporal representations, gestural stimuli involving seeing another person or having participants themselves gesture may be more effective in priming the sagittal timeline. A cognitive neuroscience approach may also be employed in future to investigate if different brain mechanisms are engaged by English and Chinese speakers when they engage in co-speech gesture processing. This is particularly relevant in light of recent research revealing language-specific co-speech gesture processing in the brain (e.g., Özyürek et al., 2007, 2008; Stevens and Zhang, 2014).

### Limitations of the present study

In addition, it is worth noting that while the dominant language of both the Singaporean English speakers and Hong Kong Chinese speakers was indeed English and Chinese respectively, there is a possibility that their bilingual background or second language acquisition and exposure may influence the results of the current cross-modal priming experiment. Future studies may investigate the role of such linguistic factors in influencing how individuals think about time, and whether the temporal concepts of bilinguals differ markedly from monolinguals.

The inherent differences between the English and Chinese words may be a source of another limitation as well. The majority of the Chinese words are adverbs as compared to the English words which comprise a mix of nouns and adverbs. As a result of this, the temporal judgments for the Chinese words might have been easier, resulting in the faster RTs of the Chinese group compared to the English group.

Another possible limitation of the present study might stem from the potential for ambiguity regarding the gestural stimuli used. Given the congruency effects observed from the English group (in addition to the absence of an anti-congruency effect, i.e., faster responding to incongruent pairs), it is unlikely that this was the case. Nonetheless, future studies that use the same paradigm may confirm this by asking participants after the experiment about whose perspective they took when understanding the actor's gestures. Steps can also be taken in a future study to rule this out as a possible reason behind the lack of congruency effects in the Chinese group by having an experiment where the gesturer faces away from the participant. In this scenario, when the actor points forward, it would unambiguously refer to a location in front of both the actor herself as well as the participant.

## Conclusions

To conclude, the present findings provide support for the view that temporal concepts in the mind reflect spatiotemporal metaphors used to represent time in speech and writing, with time being aligned along the sagittal axis. In addition, these patterns persist even when such metaphors are absent in the immediate context. While the present study has provided evidence of a sagittal timeline in English speakers, in light of the varying directions that past and future might take in Chinese, it remains to be seen if a consistent sagittal timeline can be demonstrated for speakers of the latter. In addition, the novel paradigm in which gestures may act as a means by which we may access an individual's mental timeline may provide future researchers with a way to investigate how individuals construe time without constraining them through necessitating particular modes of response, and may reveal patterns that would otherwise be obscured as a result of task demands.

## Author note

This research is based in part on MMRN's Master's thesis submitted to the National University of Singapore.

## Ethics statement

This study was carried out in accordance with the recommendations of NUS Code and Procedures on Research, NUS-IRB with written informed consent from all subjects. All subjects gave written informed consent in accordance with the Declaration of Helsinki. The protocol was approved by the “NUS-IRB” and “CUHK-SBRE.”

## Author contributions

MN: designed the study, collected the data, and analyzed the data. MN, WG, MY, CT, and WS: wrote up the manuscript.

### Conflict of interest statement

The authors declare that the research was conducted in the absence of any commercial or financial relationships that could be construed as a potential conflict of interest.

## Footnotes

^1^*Pinyin is a Romanization of Chinese characters that details how the words are pronounced and include four diacritics that denote tones. The Pinyin provided here are the pronunciations of the words in Mandarin*.

^2^Mandarin and Cantonese are both dialects of Chinese, with the most prominent difference being the pronunciation of the words. Both dialects use the same words, phrases, and metaphors.

^3^Psychology Software Tools, Inc. [E-Prime 2.0]. (2012). Retrieved from http://www.pstnet.com.

^4^Another possible limitation of the present study might stem from the potential for ambiguity regarding the gestural stimuli used. Specifically, when the actor in the video points backwards, there is the possibility that the gesture may be interpreted by the viewer as pointing to the space in front of them instead, and vice versa when the actor points forwards. To determine if this could have affected the congruency effect found in English, we tested the interaction between word temporality (past vs. future) and congruency in the mixed effects model. The interaction was not significant, *t* = 0.417, *p* = 0.68, indicating that the congruency effect can be generalized across past and future words and that ambiguity regarding perspective taking was unlikely to have affected performance. For the full table detailing the results of the mixed effects analyses with the interaction term, please refer to the Appendix.

## References

[B1] Ahrens K. Huang C. R. (2002). Time passing is motion. Lang. Linguist. 3, 491.

[B2] Alverson H. (1994). Semantics and Experience: Universal Metaphors of Time in English, Mandarin, Hindi, and Sesotho. Baltimore: Johns Hopkins University Press.

[B3] Baayen R. H. Davidson D. J. Bates D. M. (2008). Mixed-effects modeling with crossed random effects for subjects and items. J. Mem. Lang. 59, 390–412. 10.1016/j.jml.2007.12.005

[B4] Balota D. A. Yap M. J. Hutchison K. A. Cortese M. J. Kessler B. Loftis B.. (2007). The English lexicon project. Behav. Res. Methods 39, 445–459. 10.3758/BF03193014 17958156

[B5] Bartoń K. (2013). MuMIn: Multi-Model Inference. R Package Version.

[B6] Bates D. Maechler M. Bolker B. Walker S. (2014). lme4: Linear Mixed-Effects Models using Eigen and S4. R Package Version.

[B7] Boroditsky L. (2000). Metaphoric structuring: understanding time through spatial metaphors. Cognition 75, 1–28. 10.1016/S0010-0277(99)00073-6 10815775

[B8] Boroditsky L. Fuhrman O. McCormick K. (2011). Do English and Mandarin speakers think about time differently?. Cognition 118, 123–129. 10.1016/j.cognition.2010.09.010 21030013

[B9] Boroditsky L. Ramscar M. (2002). The roles of body and mind in abstract thought. Psychol. Sci. 13, 185–189. 10.1111/1467-9280.00434 11934006

[B10] Cai Q. Brysbaert M. (2010). SUBTLEX-CH: Chinese word and character frequencies based on film subtitles. PLoS ONE 5:e10729. 10.1371/journal.pone.0010729 20532192 PMC2880003

[B11] Casasanto D. Boroditsky L. (2008). Time in the mind: using space to think about time. Cognition 106, 579–593. 10.1016/j.cognition.2007.03.004 17509553

[B12] Casasanto D. Bottini R. (2014). Mirror reading can reverse the flow of time. J. Exp. Psychol. Gen. 143, 473–479. 10.1037/a0033297 23773158

[B13] Casasanto D. Jasmin K. (2012). The hands of time: temporal Gestures in English Speakers. Cogn. Linguist. 23, 643–674. 10.1515/cog-2012-0020

[B14] Chen J. Y. (2007). Do Chinese and English speakers think about time differently? Failure of replicating Boroditsky (2001). Cognition 104, 427–436. 10.1016/j.cognition.2006.09.012 17070793

[B15] Christian B. M. Miles L. K. Macrae C. N. (2012). Your space or mine? Mapping self in time. PLoS ONE 7:e49228. 10.1371/journal.pone.0049228 23166617 PMC3499549

[B16] Clark H. H. (1973). Space, time, semantics and the child, in Cognitive Development and the Acquisition of Language, ed Moore T. (New York, NY: Academic Press), 27–63.

[B17] de la Fuente J. Santiago J. Román A. Dumitrache C. Casasanto D. (2014). When you think about it, your past is in front of you how culture shapes spatial conceptions of time. Psychol. Sci. 25, 1682–1690. 10.1177/0956797614534695 25052830

[B18] Dong W. (2004). The Chinese conceptualization of time revisited. Contemp. Linguist. 6, 189–190.

[B19] Fuhrman O. Boroditsky L. (2010). Cross-Cultural differences in mental representations of time: evidence from an implicit nonlinguistic task. Cogn. Sci. 34, 1430–1451. 10.1111/j.1551-6709.2010.01105.x 21564254

[B20] Fuhrman O. McCormick K. Chen E. Jiang H. Shu D. Mao S.. (2011). How linguistic and cultural forces shape conceptions of time: English and Mandarin time in 3D. Cogn. Sci. 35, 1305–1328. 10.1111/j.1551-6709.2011.01193.x 21884222

[B21] Gentner D. Imai M. Boroditsky L. (2002). As time goes by: evidence for two systems in processing space→ time metaphors. Lang. Cogn. Process. 17, 537–565. 10.1080/01690960143000317

[B22] Kuznetsova A. Brockhoff P. B. Christensen R. H. B (2016). lmerTest: Tests in Linear Mixed Effects Models. R Package Version 2.0–33. Available online at: https://cran.r-project.org/web/packages/lmerTest

[B23] Kendon A. (1983). Gesture and Speech: How they Interact, Vol. 11. Beverly Hills: Sage Publications.

[B24] Lai V. T. Boroditsky L. (2013). The immediate and chronic influence of spatiotemporal metaphors on the mental representations of time in English, Mandarin, and Mandarin-English speakers. Front. Psychol. 4:142 10.3389/fpsyg.2013.00142 23630505 PMC3621230

[B25] Lakoff G. Johnson M. (1999). Philosophy in the Flesh: The Embodied Mind and its Challenge to Western Thought. New York, NY: Basic Books.

[B26] McNeill D. (1992). Hand and Mind: What Gestures Reveal about Thought. Chicago, IL: University of Chicago Press.

[B27] Miles L. K. Nind L. K. Henderson Z. Macrae C. N. (2010a). Moving memories: behavioral synchrony and memory for self and others. J. Exp. Soc. Psychol. 46, 457–460. 10.1016/j.jesp.2009.12.006

[B28] Miles L. K. Nind L. K. Macrae C. N. (2010b). Moving through time. Psychol. Sci. 21, 222–223. 10.1177/0956797609359333 20424050

[B29] Núñez R. Cooperrider K. (2013). The tangle of space and time in human cognition. Trends Cogn. Sci. 17, 220–229. 10.1016/j.tics.2013.03.008 23608363

[B30] Ouellet M. Santiago J. Israeli Z. Gabay S. (2010). Is the future the right time? Exp. Psychol. 57, 308. 10.1027/1618-3169/a000036 20178942

[B31] Oxford Dictionaries (2015). Oxford University Press. Available online at: http://www.oxforddictionaries.com/

[B32] Özyürek A. Kita S. Allen S. Brown A. Furman R. Ishizuka T. (2008). Development of cross-linguistic variation in speech and gesture: motion events in English and Turkish. Dev. Psychol. 44:1040. 10.1037/0012-1649.44.4.1040 18605833

[B33] Özyürek A. Willems R. M. Kita S. Hagoort P. (2007). On-line integration of semantic information from speech and gesture: insights from event-related brain potentials. J. Cogn. Neurosci. 19, 605–616. 10.1162/jocn.2007.19.4.605 17381252

[B34] Radden G. (2004). The metaphor TIME AS SPACE across languages, in Uebersetzen, Interkulturelle Kommunikation, Spracherwerb Und Sprachvermittlung–Das Leben Mit Mehreren Sprachen: Festschrift Fuer Juliane House Zum 60. Geburtstag, eds Baumarten N. Böttger C. Motz M. Probst J. Uebersetzen H. (Bochum: Aks-verlag), 225–238.

[B35] R Core Team (2012). R: A Language and Environment for Statistical Computing. Vienna: R Foundation for Statistical Computing.

[B36] Santiago J. Román A. Ouellet M. (2011). Flexible foundations of abstract thought: a review and a theory. Spatial Dimen. Soc. Thought 39–108. 10.1515/9783110254310.39

[B37] So W. C. Low A. Yap D. F. Kheng E. Yap M. (2013). Iconic gestures prime words: comparison of priming effects when gestures are presented alone and when they are accompanying speech. Front. Psychol. 4:779. 10.3389/fpsyg.2013.00779 24155738 PMC3800814

[B38] Stevens J. Zhang Y. (2014). Brain mechanisms for processing co-speech gesture: a cross-language study of spatial demonstratives. J. Neurolinguist. 30, 27–47. 10.1016/j.jneuroling.2014.03.003

[B39] Tai J. H. (1993). Conceptual structures of Chinese spatial expressions, in Papers from the Parasession on Conceptual Representations CLS29 (Chicago, IL: Chicago Linguistic Society), 347–362.

[B40] Ulrich R. Eikmeier V. de la Vega I. Fernández S. R. Alex-Ruf S. Maienborn C. (2012). With the past behind and the future ahead: back-to-front representation of past and future sentences. Memory Cogn. 40, 483–495. 10.3758/s13421-011-0162-4 22160871

[B41] Walker E. Bergen B. K. Nunez R. (2014). Investigating Spatial Axis Recruitment in Temporal Reckoning through Acoustic Stimuli and Non-spatial Responses. Center for Research in Language Technical Report, University of California, San Diego, San Diego, CA.

[B42] Wu Y. C. Coulson S. (2007). Iconic gestures prime related concepts: an ERP study. Psychon. Bull. Rev. 14, 57–63. 10.3758/BF03194028 17546731

[B43] Xu R. (2003). Measuring explained variation in linear mixed effects models. Stat. Med. 22, 3527–3541. 10.1002/sim.1572 14601017

[B44] Yap D. F. So W. C. Yap M. J. Tan Y. Q. Teoh R. L. S. (2011). Iconic gestures prime words. Cogn. Sci. 35, 171–183. 10.1111/j.1551-6709.2010.01141.x 21428996

[B45] Yu N. (2012). The metaphorical orientation of time in Chinese. J. Pragmat. 44, 1335–1354. 10.1016/j.pragma.2012.06.002

[B46] Zhou Y. Fan Y. (2015). A contrastive study of temporal-spatial Metaphor between Chinese and Americans. Theory Practice Lang. Stud. 5, 119 10.17507/tpls.0501.16

