# Supplementary material for: How We Think about Temporal Words: A Gestural Priming Study in English and Chinese
Source: Front Psychol. 2017 Jun 20;8:974. doi: 10.3389/fpsyg.2017.00974 (PMC5477416; doi:10.3389/fpsyg.2017.00974)
Supplement: Supplementary file 3 [file DataSheet1.docx]

**Appendix**

List of English temporal words

| Past-related words | |  | Future-related words | |
| --- | --- | --- | --- | --- |
| aged | memoir |  | advanced | future |
| ancestor | nostalgia |  | after | hence |
| ancient | obsolete |  | ahead | impending |
| antique | old |  | anticipated | later |
| archaic | outdated |  | approaching | looming |
| before | passe |  | brewing | modern |
| beforehand | preceding |  | coming | nearing |
| bygone | precursor |  | conclusion | next |
| classic | predecessor |  | consequently | pending |
| dated | prehistoric |  | destiny | prophesy |
| defunct | previous |  | emerging | prospective |
| departed | primitive |  | ensuing | resulting |
| earlier | prior |  | eventually | someday |
| extinct | recollection |  | expect | soon |
| flashback | reminiscence |  | fate | subsequent |
| forefather | retrospect |  | final | succeeding |
| former | stale |  | following | thereafter |
| fossilized | timeworn |  | forecast | tomorrow |
| history | vintage |  | foretell | ultimately |
| initial | yesterday |  | forthcoming | upcoming |

List of Chinese temporal words

| Past-related words | |  | Future-related words | |
| --- | --- | --- | --- | --- |
| 祖先 | 陳舊 |  | 先進 | 日後 |
| 古 | 化石 |  | 之後 | 明天 |
| 古代 | 歷史 |  | 預料 | 然後 |
| 古董 | 陳舊的 |  | 隨後 | 下一 |
| 古老 | 史前 |  | 將來 | 後續 |
| 之前 | 以前 |  | 未來 | 預測 |
| 過去 | 原始 |  | 即將 | 預言 |
| 古典 | 昨天 |  | 後來 | 此後 |
| 亡故 | 久遠 |  | 待定 | 向前 |
| 往昔 | 舊 |  | 預告 | 終究 |
| 以往的 | 回顧 |  | 預期 | 最終 |

Results of the mixed-effects analysis for the English group with the inclusion of an interaction term testing the interaction between congruency and temporality of the word.

| **English Model w/ Interaction term** |  |  |  |  |
| --- | --- | --- | --- | --- |
| **Random effects** |  |  | **Variance** | **SD** |
| Subject |  | Intercept | 0.006056 | 0.07782 |
| Item |  | Intercept | 0.002848 | 0.05337 |
| Residual |  |  | 0.013924 | 0.11800 |
| **Fixed effects** |  | **Estimate** | **SE** | ***t*-value** |
| Intercept |  | 2.975e + 00 | 3.529e-02 | 84.307*** |
| Congruency |  | -9.295-e03 | 4.184e-03 | -2.221* |
| Token duration |  | 1.903e-04 | 3.853e-05 | 4.939*** |
| Congruency*Temporality |  | 3.485e-02 | 8.364e-03 | 0.417 |
